# Relationship between Parkinson’s disease and cardio-cerebrovascular diseases: a Mendelian randomized study

**DOI:** 10.1038/s41598-023-47708-2

**Published:** 2023-11-22

**Authors:** Zhongzheng Zhou, Muzi Zhang, Qinghua Fang, Jing Huang

**Affiliations:** The Second Affiliated Hospital of Chongqing Medical University, Chongqing, China

**Keywords:** Genetics, Cardiology, Neurology

## Abstract

Parkinson’s disease (PD) and cardio-cerebrovascular diseases are related, according to earlier studies, but these studies have some controversy. Our aim was to assess the impact of PD on cardiocerebrovascular diseases using a Mendelian randomization (MR) method. The data for PD were single nucleotide polymorphisms (SNPs) from a publicly available genome-wide association study (GWAS) dataset containing data on 482,730 individuals. And the outcome SNPs data is were derived from five different GWAS datasets. The basic method for MR analysis was the inverse variance weighted (IVW) approach. We use the weighted median method and the MR-Egger method to supplement the MR analysis conclusion. Finally, We used Cochran’s Q test to test heterogeneity, MR-PRESSO method and leave-one-out analysis method to perform sensitivity analysis. We used ratio ratios (OR) to assess the strength of the association between exposure and outcome, and 95% confidence intervals (CI) to show the reliability of the results. Our findings imply that PD is linked to a higher occurrence of coronary artery disease (CAD) (OR = 1.055, 95% CI 1.020–1.091, P = 0.001), stroke (OR = 1.039, 95% CI 1.007–1.072, P = 0.014). IVW analyses for stroke’s subgroups of ischemic stroke (IS) and 95% CI 1.007–1.072, P = 0.014). IVW analyses for stroke’s subgroups of ischemic stroke (IS) and cardioembolic stroke (CES) also yielded positive results, respectively (OR = 1.043, 95% CI 1.008–1.079, P = 0.013), (OR = 1.076, 95% CI 1.008–1.149, P = 0.026). There is no evidence of a relationship between PD and other cardio-cerebrovascular diseases. Additionally, sensitivity analysis revealed reliable outcomes. Our MR study analysis that PD is related with an elevated risk of CAD, stroke, IS, and CES.

## Introduction

Parkinson’s disease (PD) is one of the most common senile diseases^1^, and its clinical manifestations are mainly resting tremors, rigidity, and bradykinesia^2^. At present, the incidence of PD is increasing worldwide^3^. Cardio-cerebrovascular diseases and it’s associated complications are also highly prevalent in older patients, and its incidence in the general elderly population is as high as 29%^4^. However, the relationship between PD and the incidence of cardio-cerebrovascular diseases is still inconclusive. Recent studies suggest that cardiovascular dysfunction may be a pre-presentation of PD, with symptoms progressively worsening as PD progresses^5^. The deposition of α-synuclein in the brain of patients with PD affects cardiac sympathetic function and leads to abnormal function of noradrenergic endings^6^. Previous epidemiological studies have also shown a possible correlation between the incidence of cardio-cerebrovascular diseases and PD^7,8^. Li Q et al. conducted a study in 2018, which showed that stroke and CAD were associated with PD in two cohorts based on Chinese populations^9^. Chua SKK et al. performed a meta-analysis showing a strong correlation between the development of PD and cardio-cerebrovascular diseases^10^. Cardio-cerebrovascular diseases are one of the leading causes of death in the elderly^11^, it is a preventable disease, and patients and those at risk can benefit from early detection and treatment^12^. We propose to use a Mendelian randomization (MR) method to assess whether there is a causal relationship between PD and cardio-cerebrovascular diseases. MR is a technique that evaluates the causal relationship between exposures and outcomes by using genetic variations that are closely related to those exposures.^13^. Since human genetic variation is randomly assigned at the time of conception, we can avoid confounding factors with this method. Furthermore, genetic variants are assigned prior to disease start, so the genotype is not influenced by the condition, reducing reverse causality^14^.

## Methods

### Mendelian randomization data and process

**Figure 1:**
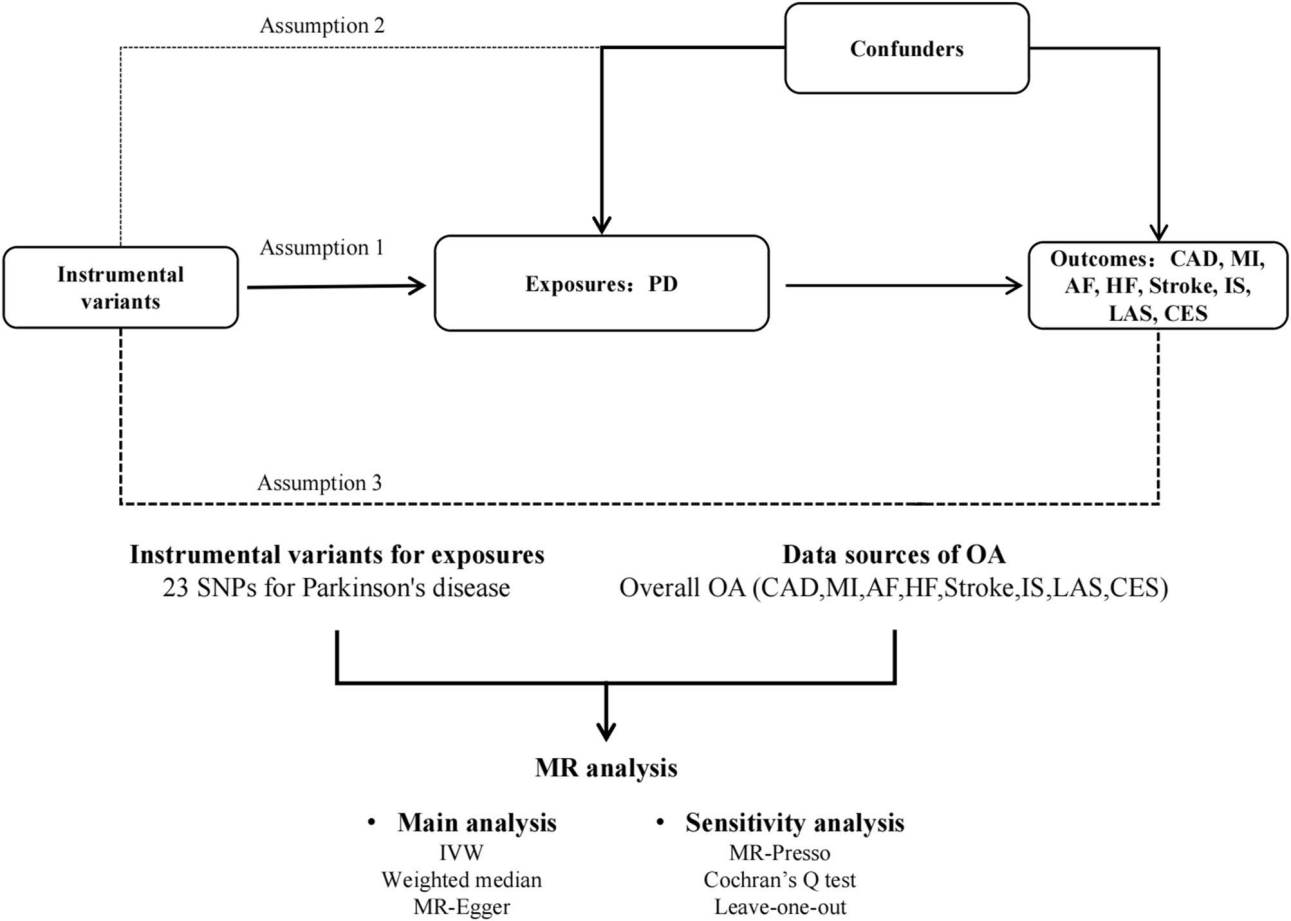
Flow chart of Mendelian stochastic analysis, The study satisfies the three main assumptions of Mendelian randomization: Assumption 1: The solid line indicates that the instrumental variants directly affects the incidence of PD. Assumption 2: Dashed lines indicate that instrumental variables are not related to any potential confounders. Assumption 3: The instrumental variables affect the outcome only through the exposure, and not through any other causal pathways.

To investigate the causal link between PD and cardio-cerebrovascular diseases, we used a two-sample MR research. Figure 1 depicts the research procedure.

### Instrumental variables selection

The SNPs included in the study must meet the three main hypotheses of MR: (i) genetic variation is strongly associated with exposure (correlation hypothesis); (ii) genetic variation is independent of known or unknown confounders (independence hypothesis); (iii) genetic variation is only associated with the outcome of exposure (exclusion hypothesis). To meet the above assumptions we developed the following inclusion criteria: The SNPs incorporated into the study were highly correlated with a significance threshold (*P* < 5 × 10^−8^) exposed to the whole genome, and all included SNPs must be in linkage equilibrium (*r*^*2*^ < 0.01). We use the F-statistic to test the significance of the results of the regression analysis; the larger the F-statistic the more significant the regression proves to be, and we consider that an F-statistic higher than 10 is needed to be strong enough to limit the bias of the weak instrumental variables, and the F-statistics was calculated as follows: F = R^2^ × (N − k − 1)/[(1 − R^2^) × k], where N refers to the sample size of GWAS for the PD, k is the numbers of SNPs, and R^2^ is the proportion of PD status explained by each SNP. Specifically, R^2^ is calculated as R^2^ = 2 × beta^2^ × (1 − EAF) × EAF, where beta2 is an estimate of the genetic effect of each SNP on PD, and EAF is the frequency of the effect allele (See Supplementary table s1 for details).

### SNPs associated with PD

The PD-related SNPs were selected from the publicly available GWAS database from International Parkinson’s Disease Genomics Consortium, which included data from a total of 482,730 European individuals, with 33,647 in the ncase group and 449,056 in the ncontrol group^15^. In total, we identified 23 loci associated with PD concentration. We used *P* < 10^–8^ as the threshold of SNPs associated with exposure, there’s no linkage disequilibrium among them, and final 23 relevant SNPs were included in the analysis. We used the F-statistic function, which describes the size and precision of the gene effect, to examine the strength of the association between SNPs and exposure factors. The calculated F-statistic is greater than 10, suggesting that the included SNPs were strongly associated with exposure.

### SNPs associated with cardio-cerebrovascular diseases

The cardio-cerebrovascular diseases included in the study were coronary artery disease (CAD), myocardial infarction(MI), atrial fibrillation (AF), heart failure (HF), stroke, ischemic stroke (IS), large artery stroke (LAS), cardioembolic stroke (CES). We show the outcome data in detail in Table 1.

**Table 1:** The basic characteristics of outcomes.

Outcomes	Consortium	Sample size (cases/controls)	Population
Coronary artery disease	Ishigaki et al.	29,319/183,134	East Asian
Myocardial infarction	CARDIoGRAMplusC4D	43,676/128,199	Mixed
Atrial fibrillation	Nielsen et al.	60,620/970,216	European
Heart failure	Shah et al.	47,309/930,014	European
Stroke	MEGASTROKE	40,585/406,111	European
Ischemic stroke	MEGASTROKE	34,217/406,111	European
Large artery stroke	MEGASTROKE	4373/406,111	European
Cardioembolic stroke	MEGASTROKE	7193/406,111	European

The SNPs for CAD were obtained from an open study by Ishigaki et al.^16^ which included 29,319 patients with CAD and 183,134 controls. The SNPs for MI were obtained from an open study by CARDIoGRAMplusC4D, which included 43,676 patients with MI and 128,199 controls^17^. The SNPs for AF were obtained from an open study by Nielsen et al.^18^ which included 60,620 patients with AF and 970,216 controls. The SNPs for HF were obtained from an open study by Shah et al.^19^ which included 47,309 patients with AF and 930,014 controls. Data on stroke and its subtypes came from the MEGASTROKE consortium^20^.

### Mendelian randomization analysis

To determine whether there is a causal association between PD and cardio-cerebrovascular diseases, we mostly employ the inverse variance weighted (IVW) approach for MR analysis. An IVW estimate is a measure of the combined overall effect size of Wald ratio estimates for individual SNPs. The IVW method is highly effective and reliable when all the SNPs included in the analysis are valid and uncorrelated^21^. We also used four other methods to perform MR analysis (the weighted median, MR-Egger, the simple mode, and the weighted mode). The weighted median estimator most effectively aggregates the effect of SNPs when the weight of valid SNPs exceeds 50%^22^. The MR-Egger regression method was employed to investigate the occurrence of genetic pleiotropy^23^. We performed a Reverse Mendelian analysis of the positive results to assess the presence of reverse causation (We describe the basic principles and algorithms of statistical methods in detail in Supplement II). We used the TwosampleMR package in R software for the entire data analysis, and the strength of association was evaluated using Odds Ratios (OR), where exposure was a risk factor for the outcome when the OR value was > 1, exposure was a protective factor for the outcome when the OR was < 1, and exposure had no effect when the OR value was = 0.

### Sensitivity analysis

To analyze horizontal pleiotropy, the intercept for MR-egger analysis was employed, and the test’s intercept does exist (*P* < 0.05), which indicates the absence of horizontal pleiotropy. To further examine the possibility of horizontal pleiotropy, we use MR-PRESSO to explore horizontal pleiotropy, which involves eliminating outliers in the data^24^. Additionally, we performed a Cochran’s Q test to screen for heterogeneity, and *P* > 0.05 demonstrated that there was none in the study’s results. To check for the possibility of MR estimate bias due to a single genetic variation, we used the leave-one-out approach. The results of the evaluation suggest that the removal of any single SNP did not significantly affect the overall results. We looked at the phenotypes database for the second phenotypes of SNPs included in the study and excluded those associated with outcome data. The detailed results of the sensitivity analysis are shown in Table 2.

**Table 2:** The results of the MR sensitivity analysis.

Outcomes	Cochran’s Q statistic	*P*-value for Cochran’s Q	*P*-value for intercept	MR-PRESSO global test
CAD	16.506	0.488	0.052	0.462
MI	19.005	0.456	0.805	0.438
AF	57.960	< 0.05	0.178	< 0.05
HF	17.681	0.279	0.401	0.316
Stroke	20.839	0.468	0.468	0.492
IS	17.029	0.709	0.876	0.711
LAS	42.679	0.003	0.074	0.006
CES	21.225	0.445	0.206	0.457

### Ethics statement

Ethical review and approval are not required for research with human participants per local legislation and institutional requirements. Written informed consent to participate is not required for this study in accordance with national legislation and institutional requirements.

## Results

### Results of the two-sample mendelian randomization analysis

In the analysis, we used 23 SNPs that had substantial associations with PD (*P* < 5 × 10^–8^), were independent of one another (R^2^ < 0.001), and were strongly linked with PD. Each SNP considered in the study had an F-statistic that was larger than 10 (F > 10), which is consistent with the first hypothesis of MR and demonstrates that the included SNPs are strongly correlated with exposure.

**Figure 2:**
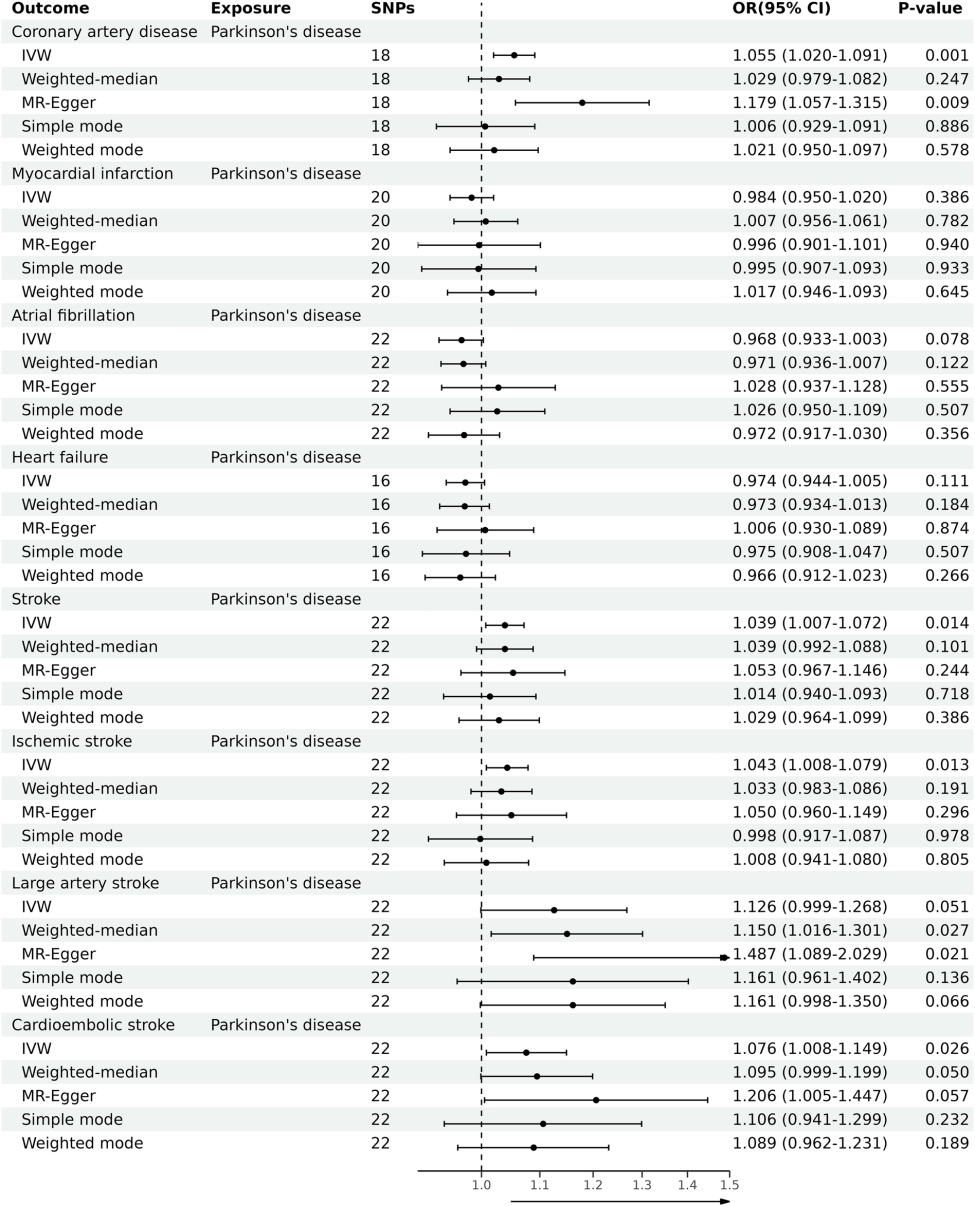
Forest plot for MR detection.

**Figure 3:**
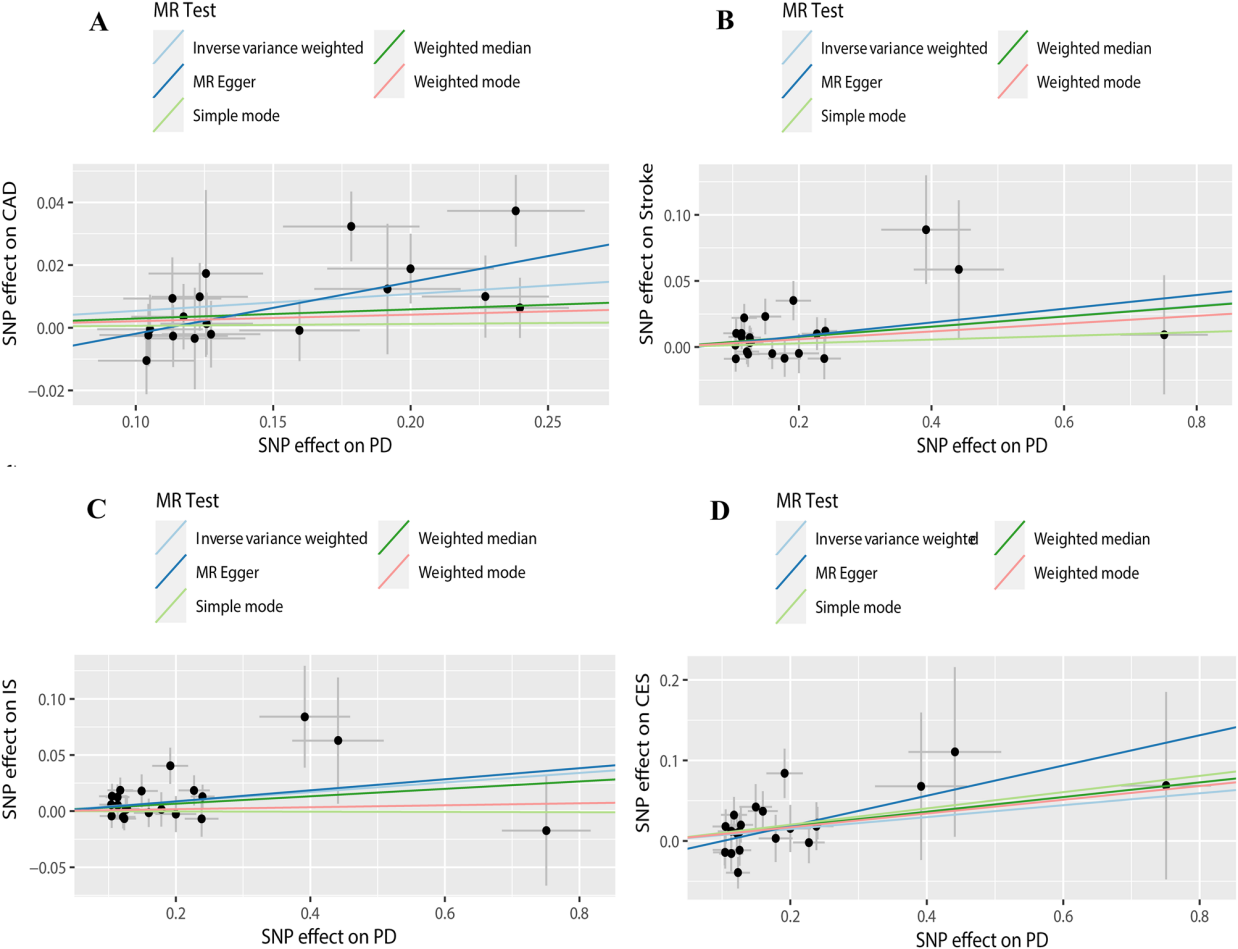
(**A**) Scatter plots of relationship between PD and CAD. (**B**) Scatter plots of relationship between PD and stroke. (**C**) A. Scatter plots of relationship between PD and IS. (**D**) Scatter plots of relationship between PD and CES.

**Figure 4:**
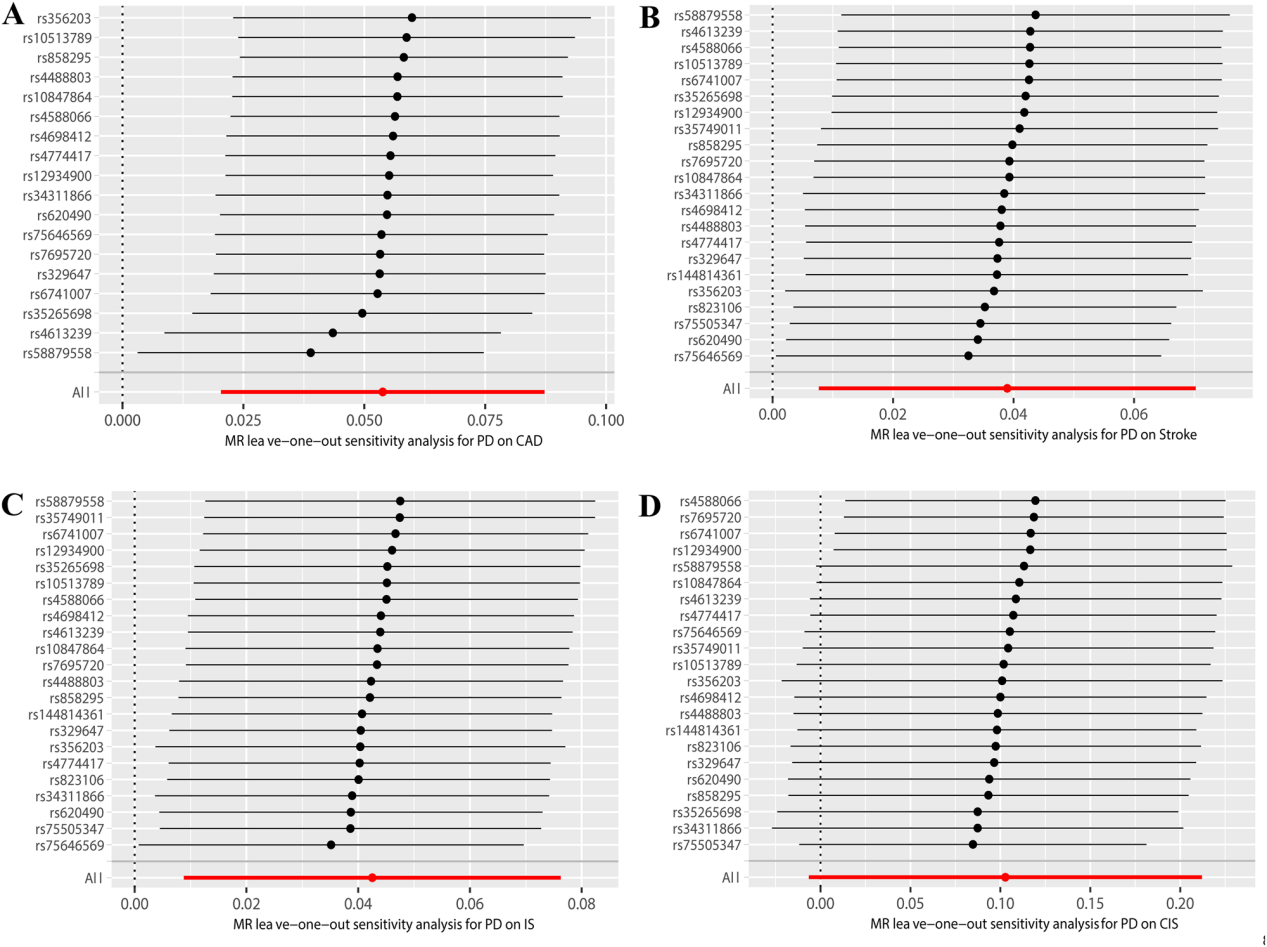
(**A**) Leave-one-out analysis between PD and CAD. (**B**) Leave-one-out analysis between PD and stroke. (**C**) A. Leave-one-out analysis between PD and IS. (**D**) Leave-one-out analysis between PD and CES.

By utilizing the merging tool to find 20 SNPs in the CADs GWAS data, we then eliminated two palindromic SNPs (rs10451230, rs823106) so that only 18 SNPs were included in the final MR analysis. According to our primary IVW analysis, PD is linked to a higher incidence of CAD (OR = 1.055, 95% CI 1.020–1.091, *P* = 0.001). The other four MR analysis methods reached similar conclusions, the weighted median (OR = 1.029, 95% CI 0.979–1.082, *P* > 0.05), the MR-Egger (OR = 1.179, 95% CI 1.057–1.315, *P* = 0.009), the simple mode (OR = 1.006, 95% CI 0.929–1.091, *P* > 0.05), and the weighted mode (OR = 1.021, 95% CI 0.950–1.097, *P* > 0.05). The forest plots of the study’s findings are displayed in Fig. 2, and the MR test scatter plots of the genetic link between PD and CAD are given in Fig. 3A. In the sensitivity analyses, there were no outliers detected with the MR-PRESSO test. The MR-Egger test and MR-PRESSO test did not find any horizontal pleiotropy across integrated SNPs (*P* > 0.05 for both tests). No heterogeneity was seen in the MR effect estimates according to the Cochran’s Q test (*P* > 0.05) (Table 2). According to the leave-one-out method, (Fig. 4A), removing a single SNP has no impact on the outcomes as a whole.

We found 23 SNPs in the stroke and its subgroups GWAS date by using the merge function and then removed one palindromic SNP (rs10451230), such that 22 SNPs were included in the final MR analysis. According to the MR test scatter plots shown in Fig. 3B, C, and D, PD is linked to an increased incidence of stroke (OR = 1.039, 95% CI 1.007–1.072, *P* = 0.014), IS (OR = 1.043, 95% CI 1.008–1.079,* P* = 0.013), and CES (OR = 1.076, 95% CI 1.008–1.147, *P* = 0.026). The results of the other four methods are as follows: The weighted median for stroke (OR = 1.039, 95% CI 0.992–1.088, *P* > 0.05), the MR-Egger for stroke (OR = 1.053, 95% CI 0.967–1.146, *P* = 0.244), the simple mode for stroke (OR = 1.014, 95% CI 0.940–1.093, *P* > 0.05), and the weighted mode for Stroke (OR = 1.029, 95% CI 0.964–1.099, *P* > 0.05); The weighted median for IS (OR = 1.033, 95% CI 0.983–1.086, *P* > 0.05), the MR-Egger for IS (OR = 1.050, 95% CI 0.960–1.149, *P* = 0.296), the simple mode for IS (OR = 0.998, 95% CI 0.917–1.087, *P* > 0.05), and the weighted mode for IS (OR = 1.008, 95% CI 0.941–1.080, *P* > 0.05); The weighted median for CES (OR = 1.095, 95% CI 0.999–1.199, *P* = 0.05), the MR-Egger for CES (OR = 1.206, 95% CI 1.005–1.447, *P* > 0.05), the simple mode for CES (OR = 1.106, 95% CI 0.941–1.299, *P* > 0.05), and the weighted mode for CES (OR = 1.089, 95% CI 0.962–1.231, *P* > 0.05). Forest plots of the study results are shown in Fig. 2. In the sensitivity analyses, there were no outliers detected with the MR-PRESSO test. We demonstrated no horizontal pleiotropy between the included SNPs. The MR effect estimates exhibited no heterogeneity according to the Cochran’s Q test (Table 2). The leave-one-out plot showed that removing specific SNPs did not significantly alter the overall effect size, as shown in (Fig. 4B, C, and D).

In our study, 20 SNPs, 22 SNPs, 16 SNPs, and 22 SNPs were included in the MR analysis of MI, AF, HF, and LAS, respectively, and the results of the IVW analyses suggested that there was no causal relationship between PD and the incidence of MI (OR = 0.984, 95% CI 0.950–1.020,* P* = 0.386), AF (OR = 0.968, 95% CI 0.933–1.003,* P* > 0.05), HF (OR = 0.974, 95% CI 0.944–1.005,* P* > 0.05), and LAS (OR = 1.126, 95% CI 0.999–1.268,* P* > 0.05). Forest plots of the study results are shown in Fig. 2, and the results of the sensitivity analysis are shown in Table 2.

### Reverse Mendelian randomization analysis

**Figure 5:**
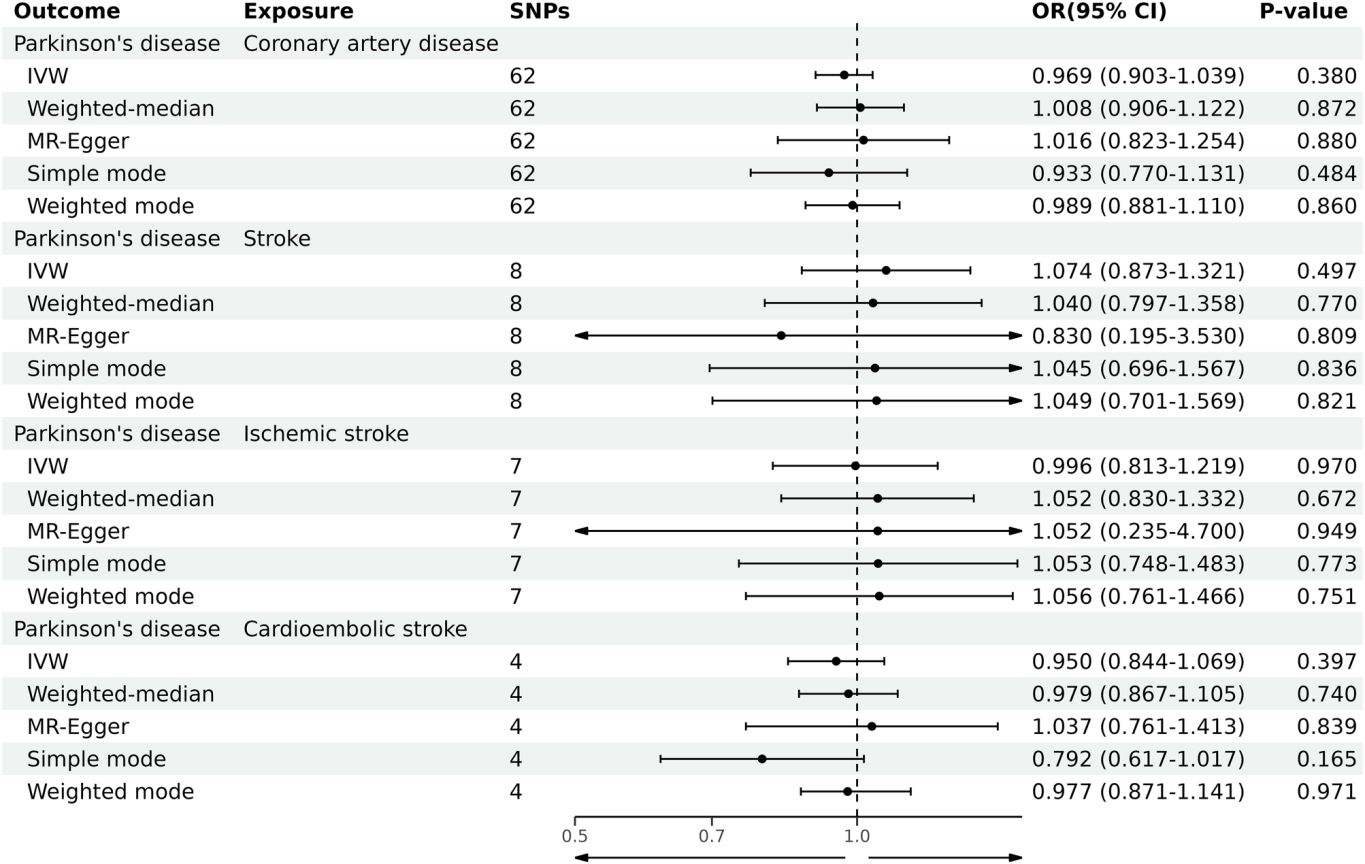
Forest plot for reverse MR detection.

We used reverse MR to see if there was a known causal association between CAD, stroke, IS, CES, and PD. The results of the IVW analyses suggested that there was no causal relationship between CAD (OR = 0.969, 95% CI 0.903–1.039,* P* = 0.380), stroke (OR = 1.074, 95% CI 0.873–1.321,* P* = 0.497), IS (OR = 0.996, 95% CI 0.813–1.219,* P* = 0.970), CES (OR = 0.950, 95% CI 0.844–1.069,* P* = 0.397) and the incidence of PD. The details of the MR analyses are shown in Fig. 5.

We performed a sensitivity analysis on the results of the reverse MR analysis (Table 3). We performed a sensitivity analysis of the results of the reverse MR analysis, in which there was horizontal pleiotropy and unreliable results in the inverse MR analysis of CAD and PD (*P* for intercept < 0.05), and there was no horizontal multiplicity or heterogeneity in the remaining three inverse MR analyses.

**Table 3:** The results of the reverse MR sensitivity analysis.

Outcomes	Cochran’s Q statistic	*P*-value for Cochran’s Q	*P*-value for intercept	MR-PRESSO global test
CAD	43.552	0.955	0.009	0.955
Stroke	3.270	0.916	0.747	0.907
IS	0.733	0.993	0.944	0.992
CES	3.393	0.334	0.601	0.407

## Discussion

Our study reveals a potential relationship between PD and CAD and stroke (including subtypes IS, CES), which is consistent with the results of previous small cohort studies, but past cohort studies were smaller and had a lower level of evidence^9,25,26^. To the best of our knowledge, this is the first study to use MR technologies to investigate the causal link between PD and cardio-cerebrovascular diseases.

PD is the second most common neurodegenerative condition after Alzheimer’s disease in terms of prevalence^27^, The main pathophysiologic mechanism of PD is the massive loss of dopaminergic neurons in dense areas of the substantia nigra; eosinophilic inclusion bodies, called Lewy bodies, appear in the cytoplasm of the remaining neurons. Because aggregated and misfolded α-synuclein is a major component of Lewy bodies, PD is classified as a synucleinopathy^28^.

We explored the relationship between PD and CAD by five MR methods, and all findings consistently suggested an association between PD and increased incidence of CAD. For the effect of PD on the increased incidence of CAD, we believe that the following mechanisms exist: First, PD is a Lewy body diseases, and excessive deposition of Lewy bodies in PD patients was clarified and reported in 1997, and excessive deposition of Lewy bodies leads to cardiac sympathetic denervation^29^. Previous autopsy analyses of deceased PD patients by researchers have revealed that the patients had significantly reduced immunoreactive tyrosine hydroxylase activity in epicardial, myocardial, and sympathetic ganglion tissues, which is thought to be closely related to Lewy body deposition in some trials^30^. Lewy body synaptic nucleoprotein lesions result in reduced sympathetic innervation of the heart and abnormal function of residual noradrenergic endings^31^, and the cardiac sympathetic denervation causes increased end-diastolic pressure in the heart, which exacerbates myocardial ischemia^32^. Second, the cardiac norepinephrine deficiency is severe in PD patients, and the loss of myocardial norepinephrine is more severe than the loss of innervation, a discrepancy that may be due to a decreased ability of nerve terminals to store catecholamines. Some studies have suggested that this results from a reduction in catecholamine vesicles caused by Lewy bodies deposition in PD patients^33^. In contrast, the reduced sensitivity to epinephrine in PD patients exacerbates symptoms of dementia, depression, bradykinesia, tremor, and ankylosis^34^, leading to a significant reduction in daily activities, and PD, as a chronic disease, may ultimately lead to the patient’s loss of mobility as the disease progresses, which greatly increases the risk of CAD^35^. Of note, orthostatic hypotension (OH) is thought to be a non-motor manifestation caused by autonomic dysfunction in PD, furthermore, OH is regarded to be closely linked to the likelihood of developing CAD^36^. Third, several studies have also pointed out that the pathogenesis of PD is closely related to oxidative stress in the nigrostriatal cells as well as systemic inflammatory response, and the above mentioned mechanisms also play an important negative impact in the pathogenesis of CAD, and these evidences suggest that there may be a common mechanism between the pathogenesis of PD and CAD^37,38^. In addition, PD also affects lipid metabolism, which in turn affects the course of CVD. Oxidized low-density lipoprotein (OxLDL) is a important contributor to cardio-cerebrovascular diseases. OxLDL competes with endothelial nitric oxide (NO) for arginine, decreasing NO bioavailability and promoting the progression of cardio-cerebrovascular diseases^39^. Several studies have found that plasma OxLDL levels are significantly higher in PD patients than in normal subjects, which may further exacerbate the risk of CVD disease^40^.

The relationship between PD and stroke has been studied more in the past, some studies have suggested that PD causes an increased risk of stroke, mainly related to abnormalities in α-synuclein metabolism, α-synuclein is a 14.5 kDa protein located predominantly in the presynaptic terminals of the mammalian brain, and over-accumulates in Lewy bodies upon gene expression^41^, some animal experiments have found a higher risk of ischemic brain injury in mice overexpressing α-synuclein than in controls^42^, and decreased expression of α-synuclein reduces cerebral infarct volume in mice^43^. α-synuclein mediates neuronal death through four main mechanisms: inflammation, oxidative stress, mitochondrial fragmentation, and autophagy, leading to an increased risk of stroke^44^. Some other studies have found that glutathione peroxidase 7 (GPX7) gene expression in PD patients is the same as in stroke patients, which may exacerbate endoplasmic reticulum oxidative stress and promote stroke^45^. In addition, OH due to PD is thought to greatly elevate the risk of IS^46^. And the link between PD and CES may be related to iron metabolism, with studies indicating that people with PD have excess iron in their brains, that excess iron creates reactive oxygen species and DNA damage^47^. Recently some MRI study found a significant causal relationship between increased iron status and CES^48^, the pathogenesis of which is that excess free iron leads to lead to the production of fibrin-like material, which leads to thrombosis^49^.

This MR study also have some limitations, first, due to racial differences in GWAS data, its test results may be affected by population stratification bias. Second, since most of the population in this study originated in Europe, we are not sure if the same conclusions can be drawn in other populations. Third, the potential pleiotropy of some SNPs may affect the detection results. Fourth, there were some sample duplications in the included studies, which could have led to biased MR estimates, but the F-statistic we detected were all greater than 10, and the assessment of this bias should be small.

In conclusion, we explored the relationship between PD and cvd at the genetic level for the first time using MR analysis, an approach that excludes environmental confounders and reverse causality bias that have been seen in previous observational studies, and provides additional clues to the pathogenesis of PD and cardio-cerebrovascular diseases as well as to therapeutic regimens. The results suggest that PD may be a potential pathogenetic factor for CAD and stroke, which is consistent with previous animal and clinical observations studies. This suggests that it is more important to achieve early screening and treatment of CAD and stroke in patients with PD.

### Supplementary Information

Supplementary Information 1.

Supplementary Table S1.

## Data Availability

The original contribution presented in this study is included in the article/supplementary material, further inquiries can be made to the corresponding author. All the datasets related to PD and cardio-cerebrovascular diseases are freely available from IEU OpenGWAS project, the link for PD is (https://gwas.mrcieu.ac.uk/datasets/ieu-b-7/), the link for CAD is (https://gwas.mrcieu.ac.uk/datasets/bbj-a-159/), the link for MI is (https://gwas.mrcieu.ac.uk/datasets/ieu-a-798/), the link for AF is (https://gwas.mrcieu.ac.uk/datasets/ebi-a-GCST006414/), the link for HF is (https://gwas.mrcieu.ac.uk/datasets/ebi-a-GCST009541/), the link for stroke and its subtypes is (https://gwas.mrcieu.ac.uk/datasets/ebi-a-GCST006906/).
