# Supplementary material for: Relationship between Parkinson’s disease and cardio-cerebrovascular diseases: a Mendelian randomized study
Source: Sci Rep. 2023 Nov 22;13:20428. doi: 10.1038/s41598-023-47708-2 (PMC10665329; doi:10.1038/s41598-023-47708-2)
Supplement: Supplementary file 1 — Supplementary Information 1. [file 41598_2023_47708_MOESM1_ESM.pdf]

Relationship between Parkinson's disease and Cardio-cerebrovascular diseases: a Mendelian randomized study

Zhongzheng Zhou[1], Muzi Zhang[1], Qinghua Fang[1], Jing Huang[1]*

Supplement II

Statistical Interpretation:

Inverse variance weighted (IVW): IVW is MR's method for summarizing effect values at multiple loci when analyzing multiple SNPs. Its arithmetic formula is as follows:

The causal effect values calculated for each genetic variant *k* for the exposure factor and the outcome variable are expressed as the following ratios:

$$Y_k / X_k$$

For the total causal effect values of all instrumental variables, they were assessed by the fixed effect model in the meta-analysis with the following equation:

$$\hat{\beta}_{IVW} = \frac{\sum_k X_k Y_k \sigma_{Yk}^{-2}}{\sum_k X_k^2 \sigma_{Yk}^{-2}}$$

The corresponding standard errors are calculated as follows:

$$se(\hat{\beta}_{IVW}) = \sqrt{\frac{1}{\sum_k X_k^2 \sigma_{Yk}^{-2}}}$$

MR-Egger: MR-Egger is based on the principle of building a linear regression model with the effect value of the genetic variable as the independent variable and the relationship between the exposure factor and the outcome variable as the dependent variable. The causal effects were estimated by the Egger regression method and tested for the presence of horizontal bias. Its arithmetic formula is as follows:

$$X_i = \sum_{j=1}^{J} \gamma_j G_{ij} + U_i + \varepsilon_i^X$$

$$Y_i = \sum_{j=1}^{J} \alpha_j G_{ij} + \beta X_i + U_i + \varepsilon_i^Y$$

$$\frac{\sum_{j=1}^{J} \hat{\gamma}_j^2 \sigma_{Y_j}^{-2} \hat{\beta}_j}{\sum_{j=1}^{J} \hat{\gamma}_j^2 \sigma_{Y_j}^{-2}}$$

$$\beta + \frac{\sum_{j=1}^{J} \gamma_j \sigma_{Y_j}^{-2} \alpha_j}{\sum_{j=1}^{J} \gamma_j^2 \sigma_{Y_j}^{-2}} = \beta + Bias(\alpha, \gamma)$$

Weighted median: The effect estimates for each SNP were ranked and the weighted median was calculated as the estimate of the causal effect. Its arithmetic formula is as follows:

$$w'_j = \frac{\hat{\gamma}_j^2}{\sigma_{Y_j}^2}$$

The simple model: Selecting a strongly associated SNP as an instrumental variable and using the effect size of this SNP to estimate the causal effect of exposure on outcome. Its arithmetic formula is as follows:

$$\hat{\beta}_{simple} = \frac{\hat{\beta}_{outcome}}{\hat{\beta}_{\exp osure}}$$

The weighted mode: The weighted mode is a method for combining the results of multiple Mendelian randomization estimates, which weights the causal effects of different genetic variants on a trait and then takes the weighted plurality as the final causal effect estimate. Its arithmetic formula is as follows:

$$\hat{\beta}_j = \frac{\hat{\Gamma}_j}{\hat{\gamma}_j}$$

Cochran's Q test: Cochran's Q test is a heterogeneity test that is used to test for significant differences between three or more groups of frequencies or rates. Its arithmetic formula is as follows:

$$Q = \sum_j Q_j = \sum_j w'_j (\hat{\beta}_j - \hat{\beta})^2$$

F-statistics: F-statistics is a common statistic in MR Studies, which is generally used to test whether the regression results are significant. Its arithmetic formula is as

follows:

$F = R^2 \times (N - k - 1) / [(1 - R^2) \times k]$

R^2 is calculated as $R^2 = 2 \times beta2 \times (1 - EAF)$ EAF, where beta2 is an estimate of the genetic effect of each SNP on PD, and EAF is the frequency of the effect allele.

References

1. Bowden J, Davey Smith G, Haycock PC, Burgess S. Consistent Estimation in Mendelian Randomization with Some Invalid Instruments Using a Weighted Median Estimator. Genet Epidemiol. 2016 May;40(4):304-14. doi: 10.1002/gepi.21965. Epub 2016 Apr 7. PMID: 27061298; PMCID: PMC4849733.

2. Bowden J, Davey Smith G, Burgess S. Mendelian randomization with invalid instruments: effect estimation and bias detection through Egger regression. Int J Epidemiol. 2015 Apr;44(2):512-25. doi: 10.1093/ije/dyv080. Epub 2015 Jun 6. PMID: 26050253; PMCID: PMC4469799.
