# Supplementary material for: Relationship between Parkinson’s disease and cardio-cerebrovascular diseases: a Mendelian randomized study
Source: Sci Rep. 2023 Nov 22;13:20428. doi: 10.1038/s41598-023-47708-2 (PMC10665329; doi:10.1038/s41598-023-47708-2)
Supplement: Supplementary file 2 — Supplementary Table S1. [file 41598_2023_47708_MOESM2_ESM.pdf]

Relationship between Parkinson's disease and cardiovascular diseases: a Mendelian randomized study

Zhongzheng Zhou, Muzi Zhang, Qinghua Fang, Jing Huang

SNP	CHR	POS	Beta	SE	P	EA	NEA	EAF	F
rs35749011	1	155135036	0.75	0.07	5.02E-30	A	G	0.02	5376
rs823106	1	205656453	-0.15	0.02	4.10E-10	C	G	0.85	2425
rs4613239	2	169119609	0.18	0.02	6.21E-13	G	C	0.13	3155
rs6741007	2	135537119	-0.12	0.02	2.09E-12	G	T	0.45	3440
rs4488803	3	58218352	-0.11	0.02	1.08E-08	A	G	0.37	2723
rs10513789	3	182760073	-0.16	0.02	3.18E-13	G	T	0.18	3206
rs7695720	4	77183300	-0.13	0.02	1.53E-09	C	A	0.21	2224
rs34311866	4	951947	0.23	0.02	7.97E-23	C	T	0.2	7191
rs4698412	4	15737348	0.13	0.02	7.05E-14	A	G	0.55	3440
rs356203	4	90666041	-0.24	0.02	3.01E-41	T	C	0.62	12150
rs75646569	5	60345424	0.19	0.03	5.62E-13	G	T	0.11	3412
rs35265698	6	32561334	-0.2	0.03	3.93E-11	G	C	0.15	4923
rs858295	7	23245569	-0.1	0.02	3.83E-09	G	A	0.39	1148
rs620490	8	16697579	-0.12	0.02	6.46E-10	G	T	0.28	2302
rs144814361	10	121410917	0.44	0.07	9.07E-11	T	C	0.02	1850
rs329647	11	133764666	-0.11	0.02	1.94E-10	C	G	0.67	2621
rs75505347	12	40885549	0.39	0.07	6.12E-09	T	C	0.02	1453
rs10847864	12	123326598	0.13	0.02	9.81E-13	T	G	0.36	3203
rs4774417	15	61993702	0.11	0.02	4.63E-08	A	G	0.74	1902
rs12934900	16	30923602	0.12	0.02	4.33E-11	T	A	0.66	3162
rs58879558	17	44095467	-0.24	0.03	1.36E-21	C	T	0.22	8764
rs10451230	17	16035225	-0.1	0.02	4.42E-08	T	A	0.57	1926
rs4588066	18	40672964	0.1	0.02	4.45E-09	A	G	0.33	2100

Supplementary table S1: Details of the PD-related Exposure data included in the study
